# Aortico-Left Atrial Fistula: A Rare Complication of Bioprosthetic Aortic Valve Endocarditis Secondary to *Enterococcus faecalis*

**DOI:** 10.1155/2015/473246

**Published:** 2015-06-28

**Authors:** Abhinav Agrawal, Martin Miguel Amor, Deepa Iyer, Manan Parikh, Marc Cohen

**Affiliations:** ^1^Department of Medicine, Monmouth Medical Center, Long Branch, NJ 07740, USA; ^2^Department of Cardiology, Robert Wood Johnson University Hospital, New Brunswick, NJ 08901, USA; ^3^Department of Cardiology, Newark Beth Israel Medical Center, Newark, NJ 07712, USA

## Abstract

Paravalvular aortic root abscess with intracardiac fistula formation is an exceedingly rare complication of infective endocarditis. This condition is even more rarely encountered in patients with bioprosthetic valve endocarditis. We report an unusual case of a 68-year-old Bosnian female with a bioprosthetic aortic valve, who developed an extensive aortic root abscess, complicated by an aortico-left atrial intracardiac fistula. This case illustrates that a high index of suspicion, prompt diagnosis by echocardiography, proper antibiotic therapy, and early surgical intervention are crucial to improving treatment outcomes for this rare condition.

## 1. Introduction

The spread of infective endocarditis from valvular structures to surrounding tissues results in periannular complications that may place patients at increased risk for adverse outcomes, including congestive heart failure, heart block, and death. Extension beyond valvular structures may result in aorto-cavitary fistulization. The incidence of this complication is estimated at 1-2% of all cases of infective endocarditis. It is seen in 3.3% of cases of prosthetic valve endocarditis and more commonly encountered with mechanical prosthetic valves compared to bioprosthetic valves [1]. We report an unusual case of a 68-year-old Bosnian female with a bioprosthetic aortic valve, who developed an extensive aortic root abscess, complicated by an aortico-left atrial intracardiac fistula.

## 2. Case Report

**Figure 1:**
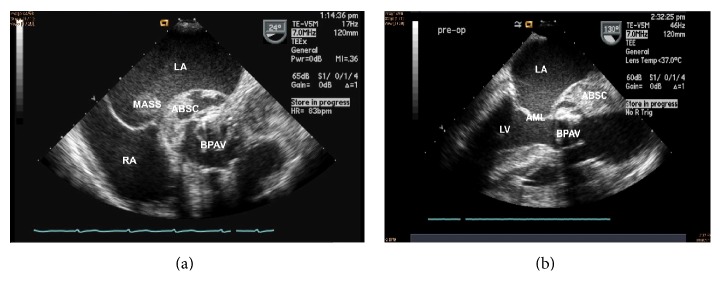
Transesophageal echocardiogram in short axis (a) and long axis (b) views, showing an extensive aortic root abscess around a bioprosthetic aortic valve, forming a fistulous communication to left atrium (legend: ABSC = abscess, LA = left atrium, MASS = mass in LA, BPAV = bioprosthetic aortic valve, RA = right atrium, AML = anterior mitral leaflet, and LV = left ventricle).

**Figure 2 fig2:**
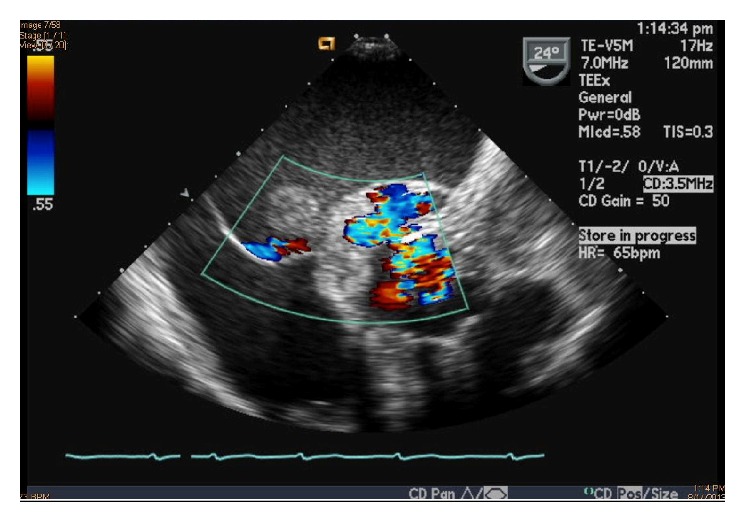
Transesophageal echocardiogram in short axis view with color flow Doppler showing blood flow from the bioprosthetic aortic valve, into the aortic root abscess, which has ruptured into the left atrium, forming an aortico-left atrial fistula.

**Figure 3:**
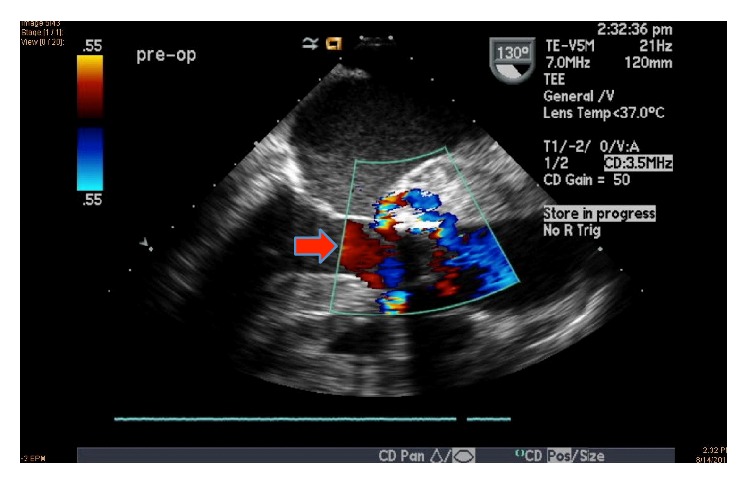
Transesophageal echocardiogram in long axis view with color flow Doppler showing severe paravalvular aortic regurgitation (indicated by red arrow), around a bioprosthetic aortic valve, around which an extensive aortic root abscess has developed.

A 68-year-old Bosnian female with prior aortic valve replacement with a bioprosthetic valve for aortic regurgitation was admitted to our facility for worsening shortness of breath, fever, and lethargy. She was on a vacation in Bosnia, where she fell ill, was hospitalized for 1 month, and was treated for suspected sepsis and renal failure. Medical records from her prior hospitalization in Bosnia were not available. She had an extensive past medical history, pertinent for coronary artery disease, diastolic congestive heart failure, atrial fibrillation, chronic kidney disease, systemic hypertension, cerebrovascular accidents, and chronic urinary tract infection (UTI). Physical examination revealed neck vein distention, bibasal crackles, and bilateral pitting edema. Auscultation revealed an irregularly irregular heart rhythm, a grade 3/6 systolic ejection murmur in the left lower sternal border, and a grade 2/6 early diastolic murmur. EKG revealed atrial fibrillation with low voltage QRS, without evidence of bundle branch blocks or conduction delays. Within an hour, she became markedly hypotensive and hypoxic. She was subsequently intubated and started on a dopamine and norepinephrine infusion. The patient was treated for septic shock with intravenous vancomycin and meropenem. Two sets of blood cultures showed growth of* Enterococcus faecalis* that was sensitive to penicillin and vancomycin. A bedside transthoracic echocardiogram (TTE) revealed new paravalvular leakage around the bioprosthetic aortic valve, raising concern for an aortic root abscess. She was transferred to a tertiary care facility for possible surgical intervention. A transesophageal echocardiogram (TEE) revealed an extensive aortic root abscess involving the entire root and base of the anterior mitral leaflet. The abscess had ruptured into the left atrium, which contained a 5–7 cm cystic mass, with a fistula connecting from the aortic root to the left atrial cavity, running through the mass. The abscess was located around the bioprosthetic aortic valve which had a large vegetation and paravalvular leak with severe paravalvular aortic regurgitation (Figures 1 [Fig fig2]–3). She became hemodynamically unstable during the TEE and was brought to the operating room for emergent surgery. She underwent homograft aortic valve replacement, aortic root replacement, VSD repair, and ligation of the aortico-left atrial fistula. Tissue samples from the excised aortic root and bioprosthetic valve also revealed growth of* Enterococcus faecalis* again found to be sensitive to penicillin and vancomycin. Postoperatively, she developed worsening septic shock. She was subsequently treated with ampicillin and gentamicin. She remained intubated and required vasopressor support with milrinone, norepinephrine, phenylephrine, and vasopressin. She became anuric and was placed on CVVHD support. Her leucocyte count as well as INR continued to increase and she progressively became more thrombocytopenic and eventually succumbed to disseminated intravascular coagulation from septic shock.

## 3. Discussion

Paravalvular extension of endocarditis is caused by bacterial destruction and invasion of local tissue and necrosis. In prosthetic valves, this process usually begins on the prosthesis cuff and often extends outside the valvular apparatus, resulting in valvular dehiscence, abscess formation, and myocardial involvement [2]. Left untreated, these abscesses may eventually rupture into adjacent cardiac chambers, leading to aorto-cavitary fistulae. Hemodynamic instability ensues due to intracardiac shunting. This leads to volume overload of the left ventricle, with subsequent left-sided heart failure and pulmonary edema.

*Staphylococcus* species are the most common bacteria implicated in aorto-cavitary fistulae, seen in 58% of cases [3]. Other bacteria implicated in the pathogenesis of this condition include* Streptococcus* spp. (28%) and* Enterococcus* spp. (7%). The remaining 7% of cases are polymicrobial. Our patient had multiple positive cultures for* Enterococcus faecalis*. Even though this bacterium is the third most common cause of infective endocarditis, it can be difficult to diagnose because the natural history of enterococcal endocarditis follows a subacute course that reminds us of other infectious and inflammatory diseases. The most common port of entry for* E. faecalis* is the urinary tract, which might also be the case in our patient suffering from chronic UTI [4].

A high index of suspicion leading to timely diagnosis and early treatment is of paramount importance in the management of aorto-cavitary fistulae. When clinically suspected, transthoracic echocardiography (TTE) is the initial technique of choice for investigation [5]. TTE is able to detect fistulous tracts in 50% of cases. This low sensitivity of detection is attributed to difficulty in characterizing abscesses at early stages, particularly when the echodensity of these lesions appears similar to contiguous tissues. With the aid of transesophageal echocardiography (TEE), overall detection rate is increased to 97%. The high rate of echocardiographic diagnosis is attributed to high-pressure differences between the aorta and the cardiac chambers. As a result of the pressure differences, flow across the fistula is highly turbulent and thus easily detectable by continuous or color Doppler monitoring. TEE also allows optimal characterization of the fistula tract, providing precise anatomic information that is invaluable for surgical planning [6]. Concern for an aortic root abscess was not entertained early on during the course of this patient's illness. This could have contributed to the delayed diagnosis of her condition.

Aortic paravalvular abscesses are associated with bundle branch blocks and first-, second-, and third-degree heart block in up to 10% of cases. These conduction defects are usually encountered when an abscess extends to the interventricular septum leading to infiltration of the nodal conduction system. Our patient did not manifest with conduction system defects on EKG. This may be explained by the course of the aorto-left atrial fistula. As shown in Figure 1, the abscess tunneled from the aortic root, posteriorly into the mitral leaflet, forming a fistulous communication with the left atrium, without infiltration of the interventricular septum.

Operative treatment remains the cornerstone of management of aorto-cavitary fistulae. Pioneering work by Ergin et al. showed that destruction and disruption of ventricular-aortic continuity in the presence of acute infective endocarditis necessitated special reconstructive techniques for treatment. Surgical treatment involved removal of all infected tissue including annular elements followed by appropriate restoration of the annulus for safe anchoring of a valve conduit [7]. Currently available conduit options for reconstruction include conventional aortic valve replacement (using a mechanical or stented biological valve), aortic valve replacement with translocation, aortic root replacement using a homograft, pulmonary autograft (Ross procedure), stentless biological valve, or a composite graft [8]. Among these options, homograft aortic root reconstruction is preferred because it offers a low recurrent infection rate and low valve-related morbidity and mortality. These aortic homografts are permeable to serum antibiotics, rendering them resistant to biofilm bacterial infection. Yankah et al. demonstrated the superiority of antibiotic-permeable cryopreserved homografts over aortic valve replacement in patient with periannular abscess [9]. The actuarial freedom from residual/recurrent infection and paravalvular leaks was 92%. Actuarial freedom from reoperation at 17 years was 75%.

Timing of operative intervention plays a pivotal role in the management of aorto-cavitary fistulae. A recent study by Kang et al. showed that as compared with conventional treatment, early surgery in patients with infective endocarditis significantly reduced mortality [10]. Our patient underwent surgical intervention after 1 month of hospitalization in Bosnia, and such delay in surgery likewise contributed to the poor outcome.

## 4. Conclusion

Aorto-cavitary fistulization is a rare and particularly problematic complication of periannular spread of infective endocarditis, with high mortality despite adequate therapy. This case illustrates that a high index of suspicion, prompt diagnosis by echocardiography, proper antibiotic therapy, and early surgical intervention are crucial to improving treatment outcomes for this rare condition.
